# Quality of Life of Children and Adolescents with Multiple Sclerosis—A Literature Review of the Quantitative Evidence

**DOI:** 10.3390/ijerph18168645

**Published:** 2021-08-16

**Authors:** Slávka Mrosková, Eleonóra Klímová, Ľudmila Majerníková, Ľubomíra Tkáčová

**Affiliations:** 1 Department of Nursing, Faculty of Health Care, University of Prešov, Partizánska 1, 08001 Prešov, Slovakia; ludmila.majernikova@unipo.sk (Ľ.M.); lubomira.tkacova@unipo.sk (Ľ.T.); 2 Department of Physiotherapy, Faculty of Health Care, University of Prešov, Partizánska 1, 08001 Prešov, Slovakia; klimova@fnsppresov.sk

**Keywords:** multiple sclerosis, quality of life, adolescent, children, school dimension of QoL, emotional dimension of QoL, disability, fatigue

## Abstract

Background: Multiple sclerosis (MS) is a chronic disease of the central nervous system that also develops in patients under 18 years of age. The disease negatively affects the quality of life (QoL) of children and adolescents. We conducted a literature review. The aim of the review was to identify the QoL of pediatric patients with MS and assess the factors determining their QoL. Methods: We analyzed studies published between 2000 and 2020 in PubMed, Scopus, Science Direct, Web of Science, and EBSCO databases. Results: In all, 17 studies were included in the review. The most common tool in assessing QoL was the generic module PedsQL. The range of mean/median global score of QoL was 53.8–81.7. The worst QoL was dominantly reported in the school and emotional spheres, on the contrary, the disease’s least determined area of QoL was the social and physical dimension. In particular, disability and fatigue were important predictors of QoL. Conclusions: MS negatively affects the school and emotional spheres in particular, so it is important to pay greater attention to these spheres of life of MS patients. As the review studies pay insufficient attention to the analysis of positive factors and their impact on the QoL of MS patients, research should integrate these phenomena. The use of MS-targeted tools in future research in the pediatric MS population is also appropriate.

## 1. Introduction

Multiple sclerosis (MS) is a chronic inflammatory disease of the central nervous system [1]. The term childhood MS is used in patients whose first clinical signs occur before 18 years of age [2]. The incidence of MS in children is rare, compared to MS in adulthood. The MS International Federation estimates that there are at least 30,000 children and teenagers with MS [3] living in the world. A recent systematic review reported an overall range of incidences from 0.05 to 2.85 per 100,000 children, and an overall prevalence from 0.69 to 26.92 per 100,000 children [4]. The slight increase in the incidence of the disease in recent years is mainly due to better and more accessible diagnostic methods but can also be attributed to a higher incidence of autoimmune diseases in developed countries [5]. Pediatric MS most commonly occurs during adolescence, and the incidence rises dramatically after puberty. Less than 1% of all pediatric MS patients have an onset before the age of 10 years. Gender ratio varies with age at onset. In prepubertal onset, the female-to-male ratio is almost equal, after puberty, the incidence of the disease in girls increases dramatically [2]. The course of the disease (95% of pediatric patients have the relapsing–remitting course), the number of relapses (higher frequency in the first years after onset of the disease), the disability (reach disability milestones at younger ages), cognitive manifestations, or the incidence of fatigue (9–56%) differs in children’s MS from adult MS [6,7,8,9,10].

Although there are different definitions or models of health-related quality of life (HRQOL), four main dimensions are incorporated: physical, mental, social, and functional health [11]. HRQOL is a subjective and multidimensional concept that makes it possible to assess the impact of the disease or pharmacological and non-pharmacological treatment on these four dimensions [12] and on well-being [13]. Identification of purpose in life and personal growth, particularly in light of a diagnosis of a progressive, chronic illness, provides a much fuller picture of an individual and their well-being and is complicated and intertwined with HRQOL [14]. Many organizations, e.g., the European Medicines Agency, emphasize the need to assess HRQOL in patients with chronic diseases [15]. The effect of the disease on the quality of life (QoL) of children has been demonstrated in various chronic diseases such as cancer [16], rheumatic diseases [17], asthma [18], diabetes mellitus [19], neurological diseases including neurosurgical patients [20], fibromyalgia [21], demyelinating diseases [22], spinal muscular atrophy [23], cerebral palsy [24], or epilepsy [25].

MS is a progressive disease with an unpredictable course that negatively affects the QoL of the patient as well as that of their family and caregivers [26,27]. Research on pediatric MS issues has grown in recent years, including studies assessing QoL. The quality of life (QoL) of adults with MS has been analyzed in a large number of patients in many countries [28,29,30]. Moreover, a recently published systematic review reported various variables affecting QoL in adult MS patients [31]. However, there is a lack of a review summarizing the studies evaluating HRQOL in pediatric MS patients and the analysis of factors that determine QoL negatively or positively.

## 2. Materials and Methods

The aim of the literature review was to analyze QoL in children and adolescents with MS and factors affecting QoL. The selection of studies respected the PRISMA guidelines. 

### 2.1. Search Strategy

The search for articles published between 2000 and 2020 was carried out in PubMed, Scopus, Science Direct, Web of Science, and EBSCO databases. The keywords used in the search were as follows: “multiple sclerosis” AND “quality of life” AND “children” (or) “adolescent” (or) “pediatric”.

### 2.2. Study Selection

The inclusion criteria for the study included the following: studies assessing QoL or QoL factors in children/adolescents diagnosed with MS, age of respondents ≤18 years, and quantitative studies written in English. The exclusion criteria were age ≥19 years, qualitative studies, review study/meta-analyses, theoretical articles, protocols, clinical therapy guidelines, and studies of validation of the tools designed to assess QoL.

**Figure 1:**
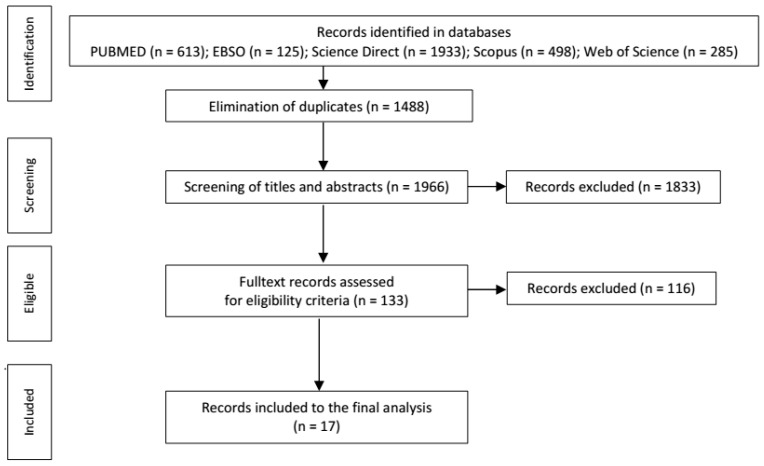
PRISMA diagram.

From the databases, we searched out 3454 sources (Figure 1). After exclusion of the duplicates (*n* = 1488), two authors of the paper (S.M., L.T.) independently carried out screening of the titles and abstracts of the contributions. In their verification, the criteria defined by the authors were respected. The criteria for the selection of contributions included the following: the ones assessing the quality of life in patients >18 years old, quality of life in case of another disease, articles of a theoretical nature/reviews, case studies, abstracts, contributions dealing with pregnant women with MS. Screening resulted in 133 articles, which were assessed in full text. At this stage, the following contributions were excluded: the ones that analyzed QoL in both pediatric and adult MS patients but did not differentiate QoL data by age, articles assessing QoL in various neurological disorders, QoL of caregivers/families, the ones that dealt with POMS (pediatric-onset multiple sclerosis) but analyzed QoL in patients >18 years, and articles about MS without quality of life assessment.

## 3. Results

In the review, we included 17 studies published between 2009 and 2020. Respondents were mostly female adolescents. The number of respondents ranged from 10 to 106. The studies were conducted in North America (*n* = 8) and European countries (*n* = 9). The study designs were mostly observational (cross-sectional or comparative) (*n* = 15) (Table 1).

### 3.1. Tools Used for the Assessment of QoL

In 15 studies [32,33,34,35,36,37,38,39,40,41,42,43,44,45,46], QoL was assessed by the generic module, PedsQL. It integrates physical and psychosocial dimensions (emotional, social, school). Furthermore, QoL was analyzed by the Child Quality of Life Child Form (TACQOL CF 12–15) [47] and the KINCREEN-52 instrument [48]. TACQOL CF 12–15 integrates six scales: pain and physical complaints, locomotor functioning, cognitive functioning, interaction with peers, and the experience of positive emotions and negative emotions; and the KIDSCREEN-52 instrument measures 10 dimensions: physical well-being, psychological well-being, moods and emotions, self-perception, autonomy, relations with parents and home life, social support and peers, school environment, social acceptance, and financial resources (Table 2). A higher score (range: 0–100) indicates a better QoL.

**Table 1:** Basic characteristics of the studies included in the literature review.

Authors	Study	Country	Sample
MacAllister et al. 2009 [32]	Cross-sectional study	USA	51 MS patients
			Age (mean ± SD, range): 14.8 ± 2.21, 9–17
			Age at onset (mean ± SD, range): 13.1 ± 2.80, 3–16
			64.7% female
			Disease duration in months (mean ± SD, range): 20.3 ± 20.4, 2–90
			EDSS (mean ± SD, range): 1.7 ± 1.5, 0–6
Mowry et al. 2010 [33]	Comparative study	USA	41 MS/CIS patients
			Age at questionnaire (mean ± SD): 14 ± 4
			62% female
			Disease duration in years (median, IQR): 2.0, 0.2–5.6
			EDSS (median, IQR): 1.5, 0–3.5
			1. Control group (*n* = 12): sibling of patients
			Age at questionnaire (mean ± SD): 13 ± 3
			75% female
			2. Control group (*n* = 38): children with neuromuscular disorders^1^
			Age at questionnaire (mean ± SD): 9 ± 5
			33% female
Lulu et al. 2014 [34]	Cross-sectional study	USA	30 MS patients
			Age (mean ± SD): 15.8 ± 2.5
			53% female
			EDSS (median, IQR): 1.5 (1–3)
Holland et al. 2014 [35]	Retrospective study	USA	26 patients (MS = 23, CIS = 3)
			Age at evaluation (mean, range): 13.96, 7–18
			65% female
			Disease duration in months (mean ± SD, range): 14.54 ± 14.38, 1–61
Yeh et al. 2017 [38]	Randomized trial	North America	52 MS patients
			Age (mean ± SD): 16.03 ± 2.20
			Age at onset (mean ± SD): 13.62 ± 2.27
			65.38% female
			EDSS (mean ± SD): 1.23 ± 1.01
Schwartzal. 2018 [39]	Randomized trial	North America	66 MS patients
			Age (mean ± SD): 15.74 ± 2.02
			Age at onset (mean ± SD):13.20 ± 3.91
			67% female
			Disease duration (mean ± SD): 2.27 ± 2.25
O’Mahony et al. 2019 [41]	Comparative study	Canada	58 MS patients
			Age at questionnaire (median, IQR): 17.0, 14.4–19.9
			Age at onset (median, IQR): 13.9, 10.9–15.1
			67.2% female
			178 monoADS patients
			Age at questionnaire (median, IQR): 12.6, 8.5–16.3
			Age at onset (median, IQR): 9.1, 4.7–12.3
			45.5% female
Marrie et al. 2020 [45]	Prospective, comparative study	Canada	36 MS patients
			Age at enrollment (mean ± SD): 13.71 ± 3.19
			Age at QoL measurement (mean ± SD): 16.61 ± 4.08
			75.0% females
			43 monoADS patients
			Age (mean ± SD): 9.67 ± 3.93
			44.2% females
			43 healthy controls
			Age (mean ± SD): 17.28 ± 4.53
			65.1% females
Ketelslegers et al. 2010 [47]	Comparative study	Netherlands	10 MS patients
			Age (mean ± SD): 15.7 ± 1.47
			Boys/girls (4/6)
			EDSS (mean ± SD): 2.5 ± 2.5
			22 monophasic patients (ADEM, NO, TM)
			Age (mean ± SD): 14.5 ± 1.78
			Boys/girls (14/8)
			EDSS (mean ± SD): 1.1 ± 1.2
			Healthy control group
Toussaint-Duysteret al. 2018 [40]	Cross-sectional, comparative study	Netherlands	22 MS patients
			Age (median, IQR): 14.0, 13.0–15.0
			Boys: 18%
			EDSS (range): 0–2, 46% at level 0, 9% at level 2
			16 ADEM patients
			Age (median, IQR): 4.5, 2.3–5.9
			Boys: 56%
			EDSS (range): 0–3, 56% at level 0, 6% at level 3
Lanzillo et al. 2016 [36]	Cross-sectional study	Italy	54 MS/CIS patients
			Age (mean ± SD): 20 ± 3.6
			52.5% female
			Disease duration in years (median): 2
			EDSS (median, range): 2.5, 0–4.5
			MSSS (mean ± SD): 5.88 ± 1.44
			Pediatric group (*n* = 34): disease onset ≤18 years
			Juvenile group (*n* = 20): disease onset 18–25 years
Ghezzi etal. 2017 [37]	Observational, prospective study	Italy	50 MS patients
			Age (mean ± SD, range): 15 ± 2.1, 12–16
			30% male sex
			EDSS (mean): 1
Ostojic et al. 2016 [48]	Retrospective, comparative study	Serbia	21 MS patients
			Age (mean ± SD, range): 16.95 ± 1.01, 14–18
			Age at onset (mean ± SD, range): 13.98 ± 2.29, 8–17.50
			71.40% female
			Disease duration in years (mean ± SD): 3.08 ± 2.50
			EDSS (mean ± SD, range): 1.71 ± 0.83, 0–3.5
			110 healthy adolescents
Johnen et al. 2019 [42]	Prospective study	Germany	19 MS patients
			Age (mean ± SD, range): 15.05 ± 2.01, 10–17
			14 females
			Disease duration in months (mean ± SD): 12.95 ± 23.52
			EDSS (mean ± SD): 0.50 ± 0.61
			Non-escalated MS group (treatment by interferon, dimethyl fumarate, glatiramer acetate) (*n* = 13)
			Escalated MS group (treatment by DMT) (*n* = 6)
Storm van’s Gravesande et al. 2019 [43]	Multicenter, cross-sectional, comparative study	Germany, Austria	106 MS patients
			Age (mean ± SD, range): 15,71 ± 1.63, 12–18
			76 females (71.7%)
			Disease duration in months (mean ± SD, range): 18.6 ± 23.7, 0–152
			EDSS (mean ± SD, range): 0.65 ± 1.09, 0–7.5
			210 healthy subjects
			Age (mean ± SD, range): 15.00 ± 2.0, 12–18
			95 females (45.2%)
Florea et al. 2020 [44]	Cross-sectional study	France	26 MS patients
			Age at onset (mean ± SD): 12.4 ± 3.1
			Age at test (mean ± SD): 15.2 ± 1.11
			Boys/girls (17/9)
			EDSS (mean ± SD): 0.1 ± 0.5
Smith et al. 2020 [46]	Retrospective study	UK	51 MS patients (QoL evaluation only in 15 patients)
			Age at onset (median): 13.7
			EDSS (median): 1

ADS—acute demyelinating syndrome. ADEM—acute disseminated encephalomyelitis. CIS—clinically isolated syndrome. IQR—interquartile range. MonoADS—monophasic acquired demyelinating syndrome. MS—multiple sclerosis. ON—optic neuritis. QoL—quality of life. TM—transverse myelitis. ^1^ Neuromuskular disorders = myopathies, muscular dystrophies, mitochondrial disorders, neuropathy/neuronopathy, central disorders of hypotonia/hypertonia, myotonia, myasthenia gravis, cerebellar ataxia.

### 3.2. Quality of Life in MS Patients

In Table 2, we present numeric values in individual QoL dimensions. The mean or median global score of QoL in MS patients ranged from 71–81.7 [33,37,41,43,46]. Only in the study of authors Lulu et al. (2014), the global score was at the median level of 53.8 [34]. The analysis of the data shows that MS adolescents have the worst QoL in the school or emotional sphere. However, up to eight studies [32,33,38,39,40,41,43,44] reported the worst QoL in the school area. In some cases, this was a decrease of up to 50–60% in QoL in this dimension. The disease least affected the social dimension of QoL [32,33,37,38,39,41,43,44,48] and the physical dimension [32,33,37,38,39,41,43,44]. The physical dimension of QoL was negatively affected by the disease to a lesser extent than the psycho-social one [34,45]. 

### 3.3. Comparison of the Quality of Life in MS Patients with a Healthy Control

MS patients showed worse QoL in all areas compared to a healthy group/healthy sibling/reference norm [32,33,43,45,47]. Ostojic et al. (2016) found lower KIDSCREEN-52 scores only in some dimensions: physical and psychological well-being, autonomy, social support and peers, school environment, and social acceptance [48].

A significant decrease in QoL in the MS group was mainly demonstrated in the physical area [32,33,45,47,48]. A significant reduction in QoL was found in social [32,47], school [32,33], cognitive [47], and emotional [32] areas.

### 3.4. Comparison of the Quality of Life in MS Patients with Other Neurological Disease Groups

MS patients reported worse QoL on all scales than monophasic patients. A significant sign was found for locomotor functioning and interaction with peers [47]. MS patients had lower scores across all dimensions of QoL than monoADS patients, except in the school district, where the mean was the same. Significant differences were only found in the emotional area [41]. Psychosocial and physical dimensions of QoL were worse in MS patients than in monoADS patients [45]. Worse QoL was reported for ADEM patients in emotional, social, and school scales compared to that in the MS group. However, MS patients had worse QoL in the physical area [40]. Compared to children with neuromuscular diseases, MS patients had better QoL in physical (*p* = 0.002) and social (*p* < 0.0001), and worse in the emotional (*p* > 0.05) and school areas (*p* = 0.049) [33].

### 3.5. Factors Determining Quality of Life

Sociodemographic factors were analyzed in four studies [32,33,37,48]. Only in the study by MacAllister et al. (2009), age of patients significantly correlated with the emotional QoL (r: −0.31) [32], and in the study by Ostojic et al. (2016), age significantly correlated with the psychological well-being (r: −0.19) and school environment (r: −0.24) [48].

As regards the clinical factors, total relapses, relapse rate, disease course, neurological abnormalities, hospitalization, physician visits, drug treatment, and non-pharmacological treatment were non-significant factors of QoL.

Disability was assessed in five studies [32-34,43,48]. All studies, except for the study by Ostojic et al. (2016), showed an impact on QoL [48]. MacAllister et al. (2009) reported that disability was correlated with the physical (r: −0.48) and social dimension (r: −0.38) [32]. An increase in the EDSS score of one point led to a 4.5 point drop in the QoL overall score [33]. Lulu et al. (2014) found that the global score of PedsQL, physical, and the psycho-social area was affected by disability [34]. Higher EDSS scores were associated with worse QoL. EDSS predicted 14.5% QoL in MS patients [43]. 

Disease duration was studied in four studies [32,33,36,48], but only Ostojic et al. (2016) showed significant correlations between disease duration and many QoL scales (physical and psychological well-being, moods and emotions, social support and peers, social acceptance) [48].

Analysis of the impact of age at disease onset suggests different outcomes. Global QoL score (*p* = 0.02) and social QoL (*p* = 0.01) were higher in pediatric than in juvenile MS patients [36], i.e., the pediatric MS patient group had a better QoL than the juvenile group. Ostojic et al. (2016) reported a positive correlation between age at the disease onset and social support and peers (r: 0.57) and social acceptance (r: 0.54), i.e., the higher age at disease onset was associated with better QoL [48].

Disease severity as a significant factor was reported by Lanzillo et al. (2016). The authors found that QoL was inversely related to MSSS in pediatric and juvenile patients [36].

The cognitive aspect of the disease was assessed in two studies [34,45]. Only Lulu et al. (2014) found that higher cognitive scores were associated with higher total QoL score [34].

Psychological factors (fatigue, anxiety, depression) were analyzed in four studies showing significant data. MacAllister et al. (2009) found that fatigue correlated with the physical (r: 0.62), emotional (r: 0.43), and school health (r: 0.62) [32]. All scales of fatigue, except for sleep fatigue, significantly correlated with PedsQL [35]. Cognitive fatigue correlated with all dimensions of QoL, the largest correlation coefficient was identified for “school functioning” (r = 0.770) and the global score of PedsQL (r: 0.658). Depression predicted 26.6% of QoL and fatigue 18.7% of QoL variation. Individuals with depression demonstrated significantly worse QoL compared to a healthy group (MS group: M = 51.84; healthy group: M= 64.44, *p* = 0.008) [43]. Ostojic et al. (2016) showed significant moderate to strong correlations between fatigue, anxiety, and depression and many physical, psycho-social, and emotional QoL scales [48] (Table 3).

## 4. Discussion

In the review studies, the QoL assessment was performed with generic tools. There are a large variety of instruments to assess the QoL in adult MS patients—for example the Multiple Sclerosis Quality of Life (MSQOL54), the Quality-of-Life Index—Multiple Sclerosis (QLI-MS), the Multiple Sclerosis Quality-of-Life Index (MSQLI), the Multiple Sclerosis International Quality-of-Life questionnaire [49]. Many of these tools have been developed to integrate the assessment of generic QoL with MS-targeted dimentions. Pediatric Neuro-QoL can be used to assess the quality of life of children with a variety of neurological diseases, including MS. This tool includes areas such as anxiety, depression, pain, cognitive function, fatigue, lower/upper extremity mobility, and stigma [50], many of which are negatively altered in children with MS shortly after onset of the disease [1,10].

According to the results of the review studies, the overall quality of life of MS adolescents was slightly reduced. Studies analyzing quality of life in adult MS patients reported different data. In some studies, QoL was significantly reduced [51,52], in others it achieved an average level [53,54,55,56], in others it was slightly altered [57]. It was also not possible to state which dimension of health was changed to a greater extent. The difference in outcomes may be related to disease duration, intensity of symptoms, severity of disease, or course of disease—for example, in patients with progressive MS, QoL has been shown to be worse compared to that of those with the relapse–remitting form [58]. Furthermore, the type of tools used in studies in adult MS patients may be related to the differences in the data presented (generic versus MS-specific tools). 

Disease in adolescents with MS most negatively affected the school and emotional area, and had the smallest impact on the social and physical area.

MS negatively affected school health. The disease course of the pediatric form of MS [59], more frequent incidence of relapses in the first years of MS diagnosis [6,7,8], increased need for hospitalizations and doctor visits [43,45,60], worse school attendance [61], cognitive impairment [61,62], and fatigue [10,43] are factors explaining the changes in school QoL, with interaction between many of these factors. Approximately 1/3 of pediatric patients have a cognitive deficiency at an early stage of the disease. Changes such as processing speed, working memory, visual–spatial processing, learning, and language [63] have important implications in outcomes of education. Fatigue is also one of the most common “invisible” symptoms in children and adolescents with MS [10,32]. Fatigue reduces attendance and concentration and causes problems with attention during the entire schoolday [64]. Carroll et al. (2019) found that tired adolescents with MS have a poorer cognitive function and higher daytime sleepiness [61]. Fatigue thus determines cognition, causing the sufferers to fall asleep during daily routine activities, which can affect adolescent’s performance at school.

Overcoming school problems requires clear communication between parents/ adolescents with MS, healthcare staff, and the school [32]. Many children and adolescents with MS have special educational needs. The possibility of using an individual educational plan, adjusting the physical space of the school, extending the time to write tests, the possibility of leaving the classroom early, etc. [65,66], are corrections that can help MS patients achieve better educational goals and thus support QoL in this area.

Emotions adversely affect adolescents with MS and negatively alter their QoL. The association between dominant depression/anxiety and QoL has been repeatedly demonstrated [43,48,67,68,69,70] and may be related to the disease itself and the specific development period—adolescence. Rainone et al. (2017) found that resilience appears to be a protective factor in QoL [70], but its positive effect decreases precisely by the action of negative emotions [71]. Therefore, specifical psychological assistance and social support [72,73,74] are very important to improve the emotional status, because the prevalence of mental problems in children with MS is high [44,75,76] and rises with the lengthening duration of the disease [77].

For adolescents, relationships with peers are of much greater importance than for children of lower age [78]. This applies to healthy children as well as sick ones [79,80,81]. The diagnosis of the disease leads to a change in interactions with peers, causing difficulty in forming relationships or testing the power of friendship. Peers provide adolescents with MS with practical and emotional support [82], which is very important to them. Communication with peers suffering from the same disease is of particular importance, as it helps with accepting the disease, sharing self-care strategies, and understanding the psycho-social dimension of the disease [66]. “Crystalizing” relationships with peers after sharing the diagnosis, achieving qualitative shifts in interactions, and establishing new relationships is a possible justification for why the social dimension of QoL in adolescents with MS is minimally altered by the disease. Social support is of great importance to chronically ill children—it has a positive impact on QoL [14], treatment adherence [83], or adjustment to the disease [84,85].

Adolescents’ relationships with parents were analyzed in the review studies minimally. The social dimension of PedsQL focuses on the quality of interactions with peers not with parents. However, Ostojic et al. (2016) showed that relationships with parents are better compared to those of healthy adolescents [48]. In healthy adolescents, there is naturally a departure from the family [78]. MS diagnosis and longer time spent with parents in the diagnostic and therapeutic process change the nature of relationships. Interactions are strengthened, relationships are closer, and adolescents share their feelings with their parents more [82].

Probably, shorter duration of the disease and low levels of disability (EDSS) in the study reviews, as well as less severe physical symptoms in children/adolescents with MS than in adult patients [7], can explain that physical QoL is only slightly affected.

Comparative studies of the review consistently point to worse QoL of adolescents with MS compared to healthy children. The results of the comparison in adult patients with MS are inconsistent. Uccelli et al. (2016) did not find significant differences in QoL between the MS population and healthy controls (QoL assessed by WHO-5), which the authors explain partially by the low level of neurological disability and the short duration of the disease in the sample [86]. Similarly, insignificant differences were reported in adult patients with MS in the physical and mental dimensions of QoL in comparison with normative data [28]. On the contrary, McCabe and McKern (2002) [87] as well as Amtmann et al. (2018) [55] demonstrated significantly worse QoL in all monitored dimensions in adult patients with MS compared to that in the general, healthy population.

QoL comparison in patients with other neurological disorders yielded mixed data. Although studies compared only the diseases of the nervous system, the course of the disease, the intensity of clinical manifestations, and the physical limitations were different in these diseases [88], which was reflected in QoL. A typical example is a study by the authors Mowry et al. (2010), which showed worse QoL in children with neurological disorders in the physical and social spheres but not in the emotional and school spheres. Concerning the type of neurological disorders (muscular dystrophies, central disorders of hypotonia/hypertonia, or myotonia) [33] and their greater negative impact on the muscle function, mobility, and education directly at school, it is logical that the physical and school QoL is worse in children with neuromuscular disorders than in MS patients.

There studies analyzed the sociodemographic, clinical, and psychological factors and their possible impact on QoL. Among the clinical factors most commonly analyzed, disability seemed to be a significant factor determining QoL, which is consistent with the studies of adult MS patients [31,54,56,57,89,90]. In a systematic review by Gil-González et al. (2020), disability was one of the most frequently significantly analyzed clinical factors in relation to quality of life [31]. Disability negatively limits a child from a variety of physical activities (sport, domestic work, and activities of daily living) or social and school activities (limiting social contacts, due to the impossibility of performing some activities with friends, and imposing restrictions on achieving educational goals), which leads to a reduction in QoL. In particular, fatigue, but also depression, and anxiety were significant QoL determinants, whereby the results for these factors were consistent and in line with adult MS patients [31]. For example, using linear regression demonstrated that depression, fatigue, and EDSS were significant predictors of QoL in adult patients [57]. Ochoa-Morales et al. (2019) reported that QoL was significantly lower in adult patients with severe depression (M = 58.13) than in patients with mild depression (M = 64.89) or patients without depression (M = 75.76) [54]. Fatigue has a negative effect not only on QoL but also on depression. Adult MS patients with a higher level of fatigue demonstrated significantly greater impairment in QoL, but also higher levels of depression [53]. Association between these factors (fatigue, depression) was reported also in pediatric MS patients [43], which multiplies their negative potential for different areas of QoL—as we pointed out in the discussion—e.g., for the school or emotional area.

## 5. Limits

The studies included in the review were mainly of an observational design (it can neither confirm nor disprove the existence of causal relationships between specific factors). Compared to studies in adult populations with MS, the variety of negative or positive factors determining QoL is significantly limited. The dominant field of review—i.e., quality of life—was analyzed using generic tools, while data on the validity and reliability of these tools for use in pediatric MS patients were missing. In comparison with a large number of MS-targeted tools for adult patients, MS-specific measure enabling the assessment of specific areas of QoL were non used in the studies. Therefore, the MS-targeted health dimensions have not been sufficiently investigated.

## 6. Conclusions

The review shows a slight decrease in the overall quality of life of MS adolescents. Since the biggest problem is the school and the emotional area of QoL, care provided to MS adolescents by schools and psychologists needs to be more cooperative. In order to improve QoL, it is necessary to assess and take into account the disability, fatigue, anxiety, and depression of each patient. Studies in the review paid insufficient attention to other factors, especially QoL protection factors such as social support, personal domains, and positive coping strategies. Therefore, QoL research in children and adolescents with MS should also investigate these factors and their impact on QoL in the future. Another gap in the detailed QoL analysis is the assessment of MS-targeted health dimensions, which requires the use of other measurement tools in research or the development and validation of a specific tool designed for the pediatric MS population.

## Figures and Tables

**Table 2:** Results of the studies included in the literature review.

Authors	Assessment of QoL	QoL in MS Group
MacAllister et al. 2009	PedsQL	Severe difficulties (score ≥ 2 SD):
		Physical scale: 20%
		Emotional scale: 10%
		Social scale: 4%
		School scale: 28%
Mowry et al. 2010	PedsQL	Mean score (SD):
		Physical scale: 73 (23)
		Psychosocial scale: 70 (17)
		Emotional scale: 65 (24)
		Social scale: 85 (15)
		School scale: 60 (23)
		Global score: 71 (17)
Lulu et al. 2014	PedsQL	Median score (IQR):
		Physical scale: 53.1 (36.3–68.8)
		Psychosocial scale: 51 (40–61.7)
		Global score: 53.8 (36.1–64.1)
Holland et al. 2014	PedsQL (used only emotional scale)	Means score (SD): 56.28 (18.79)
		Severe difficulties (score ≥2 SD): 26.9%
Yeh et al. 2017	PedsQL	Baseline mean score (SD):
		Physical scale: 82.23 (17.85)
		Emotional scale: 71.15 (20.52)
		Social scale: 83.56 (15.85)
		School scale: 66.44 (16.87)
Schwartz al. 2018	PedsQL	Mean score (SD):
		Physical scale: 80.17 (18.50)
		Emotional scale: 68.03 (23.05)
		Social scale: 83.18 (17.22)
		School scale: 63.56 (18.50)
O’Mahony et al. 2019	PedsQL	Median score (IQR):
		Physical scale: 87.5 (75.0–93.8)
		Psychosocial scale: 80.0 (65.0–90.0)
		Emotional scale: 75.0 (55.0–90.0)
		Social scale: 90.0 (80.0–100.0)
		School scale: 75.0 (56.7–90.0)
		Global score: 81.5 (73.9–91.3)
Marrie et al. 2020	PedsQL	Mean score (SD):
		Psychosocial scale: 76.13 (15.50)
		Physical scale: 81.14 (19.49)
Ketelslegers et al. 2010	TACQOL CF 12–15	-
Toussaint-Duysteret al. 2018	PedsQL	Impaired QoL in MS group (score of 1 SD below the mean of healthy age-related reference norm):
		Physical scale: 45%
		Emotional scale: 18%
		Social scale: 32%
		School scale: 46%
		Global score: 41%
Lanzillo et al. 2016	PedsQoL	-
Ghezzi et al. 2017	PedsQL	Baseline mean score (SD):
		Physical scale: 81.3 (15.9)
		Emotional scale: 73.1 (17.9)
		Social scale: 90.3 (13.3)
		School scale: 75.6 (18.5)
		Psychosocial scale: 79.7 (13.8)
		Global score: 80.3 (13.5)
Ostojic et al. 2016	KIDSCREEN-52	Mean score (SD):
		Physical well-being: 47.00 (11.25)
		Psychological well-being: 49.82 (12.76)
		Moods and emotions: 51.65 (12.48)
		Self-perception: 50.74 (10.19)
		Autonomy: 53.40 (10.88)
		Parent relation and home life: 55.12 (9.68)
		Social support and peers: 52.91 (13.55)
		School environment: 47.65 (10.24)
		Social acceptance: 50.86 (12.67)
		Financial resources: 51.96 (8.70)
Johnen et al. 2019	PedsQL (psychosocial scale = emotional, social, school scale)	Baseline mean (SD) psychosocial scale in non-escalated: 4.50 (14.00), and in escalated group: 12.00 (13.00)
Storm van’s Gravesande et al. 2019	PedsQL	Mean score (SD):
		physical scale: 74.62 (22.1)
		emotional scale: 63.35 (24.89)
		social scale: 88.73 (17.01)
		school scale: 58.15 (24.74)
		global score: 71.81 (18.36)
Florea et al. 2020	PedsQL	Poor QoL (below ≥75 points):
		physical scale: 20%
		emotional scale: 50%
		social scale: 5%
		school scale: 50%
		global score: 40%
Smith et al. 2020	PedsQL	Median global score (IQR): 81.7 (65.3–92.4)

PedsQL—Pediatric Quality of Life Inventory. QoL—quality of life. TACQOL CF 12–15—Child Quality of Life Child Form 12–15. IQR—interquartile range. SD—standard deviation.

**Table 3:** Factors analyzed in relation to quality of life.

Authors	Non-Significant Factors	Significant Factors
MacAllister et al. 2009	Disease duration	Age
	Total relapses. Relapse rate	Disability (EDSS)
	Age at onset disease	Fatigue (MFS)
Mowry et al. 2010	Age. Sex. Race. Ethnicity.	Disability (EDSS)
	Drug therapy (DMT). Disease duration	
Holland et al. 2014	Sleep fatigue	Fatigue—general, cognitive, emotional, total (MFS)
Lulu et al. 2014	Treatment adherence	Disability (EDSS)
		Cognitive score (SDMT)
Lanzillo et al. 2016	Disease duration	Disease severity (MSSS)
		Age at onset disease
Ostojic et al. 2016	Number of relapses.	Age
	Disability (EDSS)	Age at disease onset. Disease duration
		Anxiety and depression (RCADS)
		Fatigue (PedsFACIT-F)
Ghezzi et al. 2017	Age. Sex	-
	Age at onset disease.	
	Disease severity.	
	Drug treatment (interferon-β1).	
	Treatment naïveness	
Yeh et al. 2017	-	Non-pharmacologic interventions
O’Mahony et al. 2019	Disease severity	-
Storm van’s Gravesande et al. 2019	Disease course (acute relapse, remision)	Disability (EDSS)
		Cognitive fatigue (MFS)
		Depression (Depressionstest fur Kinder—DTK, German self-report questionnaire, BDI)
Johnen et al. 2019	Drug treatment	-
Marrie et al. 2020	Number of relapses. Hospitalization.	-
	Physician visits.	
	Neurologic abnormality	
	(neurological examination).	
	Cognitive accuracy/cognitive response time (Penn Neurocognitive Battery)	

BDI—Beck Depression Inventory. DMT—disease-modifying therapy. EDSS—Expanded Disability Status Scale. MFS—Multidimensional Fatigue Scale. MSSS—Multiple sclerosis severity score. PedsFACIT-F—Pediatric–Functional Assessment of Chronic Illness Therapy—Fatigue. RCADS—Revised Child Anxiety and Depression Scale. SDMT—Symbol Digit Modalities Test.

## Data Availability

No additional data available.
